# Predictive ability of EuroSCORE II integrating cardiactroponin T in patients undergoing OPCABG

**DOI:** 10.1186/s12872-020-01745-1

**Published:** 2020-10-28

**Authors:** Xiang Li, Lingtong Shan, Mengwei Lv, Zhi Li, Chunyan Han, Ban Liu, Wen Ge, Yangyang Zhang

**Affiliations:** 1 Department of Cardiology, The First Affiliated Hospital of Chongqing Medical University, Chongqing Medical University, Chongqing, People’s Republic of China; 2 Department of Thoracic Surgery, Sheyang County People’s Hospital, Yancheng, Jiangsu People’s Republic of China; 3 Department of Thoracic Surgery, Yancheng Third People’s Hospital, The Affiliated Yancheng Hospital of Southeast University Medical College, Yancheng, Jiangsu People’s Republic of China; 4 Shanghai East Hospital of Clinical Medical College, Nanjing Medical University, Shanghai, People’s Republic of China; 5 Department of Cardiovascular Surgery, Shanghai East Hospital, Tongji University School of Medicine, 150 Jimo Road, Shanghai, 200120 People’s Republic of China; 6 Department of Cardiovascular Surgery, Jiangsu Province Hospital, The First Affiliated Hospital With Nanjing Medical University, Nanjing, Jiangsu People’s Republic of China; 7 Department of Cardiology, Shanghai Tenth People’s Hospital, Tongji University School of Medicine, No.301 Central Yanchang Road, Shanghai, 200072 People’s Republic of China; 8 Department of Cardiothoracic Surgery, Shuguang Hospital, Affiliated to Shanghai University of TCM, Shanghai, People’s Republic of China

**Keywords:** EuroSCORE II, Cardiac troponin t, Coronary artery bypass grafting, Off-pump, Mortality

## Abstract

**Background:**

Preoperative risk evaluation systems are significant and important to the allocation of medical resources and the communication between doctors and patients. The European System for Cardiac Operative Risk Evaluation II (EuroSCORE II) is widely used in clinical practice. Cardiac troponin T (cTnT) can specifically and accurately reflect myocardial injury. Whether EuroSCORE II can improve the predictive power after integrating with cTnT is still unclear. This study was a retrospective single center study designed to assess the predictive ability of EuroSCORE II integrated with cTnT for patients undergoing isolated off-pump coronary artery bypass grafting (OPCABG).

**Methods:**

This retrospective and observational cohort study included 1887 patients who underwent first isolated OPCABG. cTnT was detected within 48 h before operation in each patient. According to myocardial injury, patients were divided by cTnT into 4 stages. A new risk evaluation system was created through logistic regression with EuroSCORE II and myocardial injury classification as covariates. Then the two risk evaluation systems were comparatively assessed by regression analysis, receiver operator characteristic curves, net reclassification index, Bland–Altman plots and decision curve analysis.

**Results:**

There were 43 in-hospital deaths, with a mortality of 2.30% (43/1887). The logistic regression analysis showed that preoperative myocardial injury classification was a significant risk factor for in-hospital mortality in both total cohort (OR 1.491, 95%CI 1.049–2.119) and subsets (OR 1.761, 95%CI 1.102–2.814). The new risk evaluation system has higher calibration and discrimination power than EuroSCORE II, both for overall cohort and subsets. Especially, the new system has obvious advantages in discrimination power in the subset of acute myocardial infarction (AUC 0.813 vs. 0.772, 0.906 vs. 0.841, and 0.715 vs. 0.646, respectively).

**Conclusions:**

Both myocardial injury classification and EuroSCORE II are independent risk factors of in-hospital mortality in OPCABG patients. The new risk evaluation system has higher predictive ability than EuroSCORE II, especially in patients with a recent history of AMI.

## Background

The number of cardiac surgery is increasing rapidly in China following the development of economy and surgical technology [1]. In the past two decades, the proportion of coronary artery bypass grafting (CABG) has been rising fast, more than half of which is off-pump CABG (OPCABG) in mainland China [2]. Preoperative risk evaluation is important and significant and can help surgeons to judge the diagnosis and treatment of potential postoperative complications. The European System for Cardiac Operative Risk Evaluation II (EuroSCOREII) is one of the commonly- and widely-used risk evaluation systems in clinical practice. CABG is a special operation of myocardial revascularization for patients suffering myocardial ischemia injury to different degrees before operation. Myocardial markers, such as cardiac troponin T (cTnT), can specifically and accurately reflect the situation of myocardial injury [3, 4]. However, EuroSCOREII does not include myocardial markers as important variables. Therefore, we suspect whether EuroSCOREII can improve the predictive power after integrating with cTnT for patients undergoing OPCABG. As far as we know, there are only a few reports on this issue [5–7]. Hence, this study was aimed to comparatively evaluate the predictive ability of EuroSCOREII integrating with cTnT in OPCABG patients.

## Methods

### Patients

Between January 2010 and October 2017, 1902 consecutive patients underwent primary isolated OPCABG in our department. Exclusion criteria were preoperative hemodialysis, lack of cTnT before operation, and incomplete medical records. Finally, 1887 patients made up the study database. All included patients, or their legal representatives, signed written informed consents to take part in the study and for all surgical procedures. We reviewed and collected detailed clinical data from medical records and the hospital information system.

### Data collection

Venous blood (within 48 h before operation) of each patient was collected and sent to cTnT measurement by standard techniques in the central laboratory of our hospital. Since January 2015, the new-generation high-sensitivity cTnT (hs-cTnT) has been widely used in clinical practice. The detection limits of cTnT and hs-cTnT are 100—2000 and 0—10,000 ng/l, respectively, and their normal ranges are 0—100 and 0—14 ng/l respectively. The risk score of each patient was calculated online according to the EuroSCOREII interactive calculator (https://www.euroscore.org/calcold.html). Myocardial injury classification was divided by the preoperative cTnT into stages 1 to 4, in which the preoperative cTnT is within the normal range, above the upper normal limit (UNL) but less than 10 times of UNL, above 10 times of UNL but less than 20 times of UNL, and above 20 times of UNL respectively.

### Research registration

All patients were operated by the same group of surgeons. After operation, treatment and nursing care were performed in accordance with the routine of the department and clinical guidelines. The study was approved by the Ethics committees of the local hospital (ID: 2017–018). The trial was registered at https://www.chictr.org.cn with NO.ChiCTR2000032365.

### Creation of new model

Multivariable logistic regression with backward elimination was modeled by using the variables from univariate analysis to identify the independent risk factors associated with in-hospital mortality. All variables with *P < *0.15 or clinically considered as risk factors were entered into the multivariate analysis, including gender, age, New York Heart Association (NYHA) stage, cerebrovascular disease, creatinine clearance rate (Ccr), left ventricular ejection fraction (LVEF), peripheral vascular disease, operation status, renal dysfunction, EuroSCORE II, myocardial injury classification, diabetes, valvular disease, body mass index (BMI) and hypertension. After indemnifying EuroSCORE II and myocardial injury classification as independent risk factors for in-hospital mortality, we used these two factors as covariates to create a new risk evaluation system by logistic regression.

### Statistical analysis

Continuous data was expressed as mean ± standard deviation or median and interquartile ranges (non-normal distribution), and were compared between groups through Student’s t test or Mann–Whitney U test. Categorical variables were presented as number and percentage and were compared using Fisher’s exact or Chi-square tests.

The new risk evaluation system and EuroSCORE II were calibrated by Hosmer–Lemeshow (H–L) goodness-of-fit statistic. Discrimination ability was measured by receiver’s operating characteristic (ROC) curve. Discrimination power was considered by the area under the curve (AUC) of ROC.

Agreement between the two risk evaluation systems was estimated by Bland–Altman analysis [8]. The predictive in-hospital mortality was calculated by the two systems separately. Figures were plotted by the differences between the two sorts of predictive mortalities and the mean between them. All differences being equal to 0 indicate the two systems fully agree with each other. But certain error always existed in the risk evaluation systems. Patients who died and those who survived were sent to Bland–Altman analysis separately. A better risk evaluation system will give higher predictive mortality in dead patients and lower predictive mortality in surviving patients. The agreement interval was calculated using the mean of differences ± standard deviation. Over 95% of the points fell in the agreement interval, which indicated a good agreement between the two risk evaluation systems.

The net benefits of the two risk evaluation systems for predicting in-hospital mortality were measured by decision curves analysis (DCA). As reported [9], the proportion of all false positive patients was subtracted by DCA from the proportion of true positive patients, and then weighted according to the relative harm of false positive and false negative results.

The consistency of the two evaluation systems in predicting in-hospital mortality was tested by the net reclassification index (NRI). According to Pencina et al. [10], patients were divided into 4 groups by different standards. A change into a higher group means upward movement (up), and a change into a lower group means downward movement (down). The NRI was calculated as follows:

$$NRI=P\left(\frac{up}{down}\right)-p\left(\frac{down}{event}\right)+P\left(\frac{down}{nonevent}\right)-P\left(\frac{up}{nonevent}\right)$$

Statistical analysis was performed on SPSS 22.0 for windows (IBM, Chicago, USA). DCA was performed on R software 3.4.0 with the package Decision curve. Two-sided *P *≤ 0.05 was considered significant.

### Outcome endpoint

The endpoint of this study was in-hospital mortality, which was defined as any death within 30 days after operation or during postoperative hospitalization.

## Results

### Demographic data

A total of 1887 patients were divided by cTnT into a cTnT group (n = 971) and a hs-cTnT group (n = 916). Baseline clinical characteristics and demographics of the patients were shown in Table 1. Patients in the cTnT group had more frequent preoperative comorbidities, such as hypertension, and unstable angina. Patients in the cTnT group had significantly higher Cr and lower Ccr than the hs-cTnT group (both *P < *0.001). The NYHA stage, LVEF and EuroSCORE II were all higher in the cTnT group than the hs-cTnT group.

**Table 1:** Demographics and clinical characteristics of total cohort and subsets

Variables	Total (n = 1887)	cTnT group (n = 971)	hs-cTnT group (n = 916)	*P*
Age (y)	64.94 (60;71)	65.15 (60;71)	64.70 (60;71)	0.413
Female (n, %)	460 (24.4)	236 (24.3)	224 (24.5)	0.940
Weight (kg)	68.88 (61;69)	69.14 (61;76)	68.61 (60;75)	0.115
Height (cm)	166.52 (160;172)	166.53 (160;172)	166.51 (160;172)	0.851
BMI (kg/m^2^)	24.79 (22.83;26.67)	24.89 (22.86;26.85)	24.69 (22.66;26.52)	0.066
BSA (m^2^)	1.74 (1.63;1.85)	1.75 (1.63;1.86)	1.74 (1.62;1.84)	0.226
Diabetes (n, %)	590 (31.3)	324 (33.4)	266 (29.0)	0.043
Hypertention (n, %)	1281 (68.4)	722 (74.4)	569 (62.1)	0.000
Cr (μmol/l)	82.44 (64.80;90.10)	83.99 (68.00;91.90)	80.79 (62.43;86.00)	0.000
Ccr (mL/min/1.73 m^2^)	80.87 (63.31;95.83)	78.69 (62.17;93.39)	83.16 (64.86;99.32)	0.000
Renal failure (n, %)	29 (1.5)	11 (1.1)	18 (2.0)	0.236
Myocardial injury classification				0.212
Stage 1 (n, %)	1322 (70.1)	894 (92.1)	428 (46.7)	
Stage 2 (n, %)	455 (24.1)	66 (6.8)	389 (42.5)	
Stage 3 (n, %)	45 (2.4)	6 (0.6)	39 (4.3)	
Stage 4 (n, %)	65 (3.4)	5 (0.5)	60 (6.6)	
Cerebrovascular disease (n, %)	350 (18.5)	254 (26.2)	96 (10.5)	0.100
COPD (n, %)	54 (2.9)	21 (2.2)	33 (3.6)	0.311
Peripheral vascular disease (n, %)	47 (2.5)	30 (3.1)	17 (1.9)	0.517
Previous PCI (n, %)	115 (6.1)	31 (3.2)	84 (9.2)	0.296
Atrial flutter and fibrillation (n, %)	66 (3.5)	34 (3.5)	32 (3.5)	0.385
Combined valvular disease (n, %)	249 (13.2)	71 (7.3)	178 (19.4)	0.001
Pulmonary hypertension (n, %)	179 (9.5)	21 (2.2)	158 (17.2)	0.177
Types of CAD				0.000
AMI (n, %)	260 (13.8)	161 (16.6)	99 (10.8)	
Unstable angina (n, %)	1028 (54.5)	567 (58.4)	461 (50.3)	
Number of diseased vessels (n)	2.85 (3;3)	2.85 (3;3)	2.85 (3;3)	0.459
NYHA				0.002
I (n, %)	217 (31.7)	67 (6.9)	150 (16.4)	
II (n, %)	1229 (65.1)	684 (70.4)	545 (59.5)	
III (n, %)	404 (21.4)	202 (20.8)	202 (22.1)	
IV (n, %)	37 (2)	18 (1.9)	19 (2.1)	
LVEF (%)	60.92 (59.9;65.4)	61.75 (60.0;65.9)	60.03 (58.0;65.2)	0.000
EuroSCORE II	1.68 (0.94;2.03)	1.82 (1.01;2.22)	1.53 (0.85;1.80)	0.000
Bypass graft number (n)	3.54 (3;4)	3.54 (3;4)	3.55 (3;4)	0.444
In-hospital mortality (n, %)	43 (2.3)	21 (2.2)	22 (2.4)	0.155

hs-cTnT, high sitivity cardiac troponin T; cTnT, cardiac troponin T; BMI, body mass index; BSA, body surface area; Cr, creatinine; Ccr, creatinine clearance rate; COPD, chronic obstructive pulmonary disease; PCI, percutaneous coronary intervention; CAD, coronary artery disease; AMI, acute myocardial infarction; NYHA, New York Heart Association; LVEF, left ventricular ejection fraction; EuroSCORE, European system for cardiac operative risk evaluation. # comparison between cTnt group and hs-cTnT group

The degree of myocardial injury significantly differs with the type of coronary heart disease (CAD) [11]. The total cohort was divided by the classification of clinical CAD into 3 subsets, including stable angina, unstable angina, and acute myocardial infarction (AMI). Demographics and perioperative data were revealed in Table 2.

**Table 2:** Demographics and clinical characteristics of groups classified by type of CAD

Variables	Stable group (n = 599)	Unstable group (n = 1028)	AMI group (n = 260)	*P*
Age (y)	64.62 (59;71)	65.34 (61;71)	64.06 (59;70)	0.052
Female (n, %)	119 (19.9)	278 (27.0)	63 (24.2)	0.005
Weight (kg)	69.45 (62;76)	68.75 (60;75)	68.11 (61;74)	0.154
Height (cm)	167.12 (162.00;172.00)	166.15 (160.00;171.00)	166.60 (160.25;171.75)	0.053
BMI (kg/m^2^)	24.83 (22.89;26.84)	24.85 (22.84;26.77)	24.49 (22.54;25.92)	0.105
BSA (m^2^)	1.76 (1.64;1.87)	1.74 (1.62;1.85)	1.74 (1.63;1.83)	0.155
Diabetes (n, %)	149 (24.9)	352 (34.2)	89 (34.2)	0.000
Hypertention (n, %)	364 (60.8)	743 (72.3)	184 (70.8)	0.000
Cr (μmol/l)	83.25 (64.60;90.20)	81.82 (64.50;89.58)	83.02 (67.78;91.73)	0.164
Ccr (mL/min/1.73 m^2^)	82.28 (65.12;91.06)	81.12 (62.53;95.94)	79.14 (62.35;90.86)	0.076
Renal failure (n, %)	7 (1.2)	17 (1.7)	5 (1.9)	0.642
Myocardial injury classification				0.000
Stage 1 (n, %)	414 (69.1)	767 (74.6)	141 (54.2)	
Stage 2 (n, %)	160 (26.7)	217 (21.1)	78 (30.0)	
Stage 3 (n, %)	9 (1.5)	17 (1.7)	19 (7.3)	
Stage 4 (n, %)	16 (2.7)	27 (2.6)	22 (8.5)	
Cerebrovascular disease (n, %)	81 (13.5)	221 (21.5)	48 (18.5)	0.000
COPD (n, %)	18 (3.0)	27 (2.6)	9 (3.5)	0.746
Peripheral vascular disease (n, %)	17 (2.8)	23 (2.2)	7 (2.7)	0.736
Previous PCI (n, %)	22 (3.7)	76 (7.4)	17 (6.5)	0.010
Atrial flutter and fibrillation (n, %)	14 (2.3)	40 (3.9)	12 (4.6)	0.148
Combined valvulardisease (n, %)	138 (23.0)	67 (6.5)	44 (16.9)	0.000
Pulmonary hypertension (n, %)	69 (11.6)	83 (8.1)	27 (10.4)	0.063
Number of diseased vessels (n)	2.84 (3;3)	2.85 (3;3)	2.87 (3;3)	0.683
NYHA				0.000
I (n, %)	68 (11.4)	109 (10.6)	40 (15.4)	
II (n, %)	402 (67.1)	691 (67.2)	136 (52.3)	
III (n, %)	122 (20.4)	212 (20.6)	70 (26.9)	
IV (n, %)	7 (1.2)	16 (1.6)	14 (5.4)	
LVEF (%)	61.45 (60.10;65.60)	61.32 (60.00;65.78)	58.09 (52.27;64.40)	0.000
EuroSCORE II	1.44 (1.21;1.79)	1.73 (0.95;2.10)	2.02 (0.96;2.47)	0.000
Bypass graft number (n)	3.69 (3;4)	3.44 (3;4)	3.60 (3;4)	0.000
In-hospital mortality (n, %)	14 (2.3)	19 (1.8)	10 (3.8)	0.155

CAD, coronary artery disease; MI, myocardial infarction; BMI, body mass index; BSA, body surface area; Cr, creatinine; Ccr, creatinine clearance rate; COPD, chronic obstructive pulmonary disease; PCI, percutaneous coronary intervention; NYHA, New York Heart Association; LVEF, left ventricular ejection fraction; EuroSCORE, European system for cardiac operative risk evaluation

### Perioperative outcomes

**Fig. 1:**
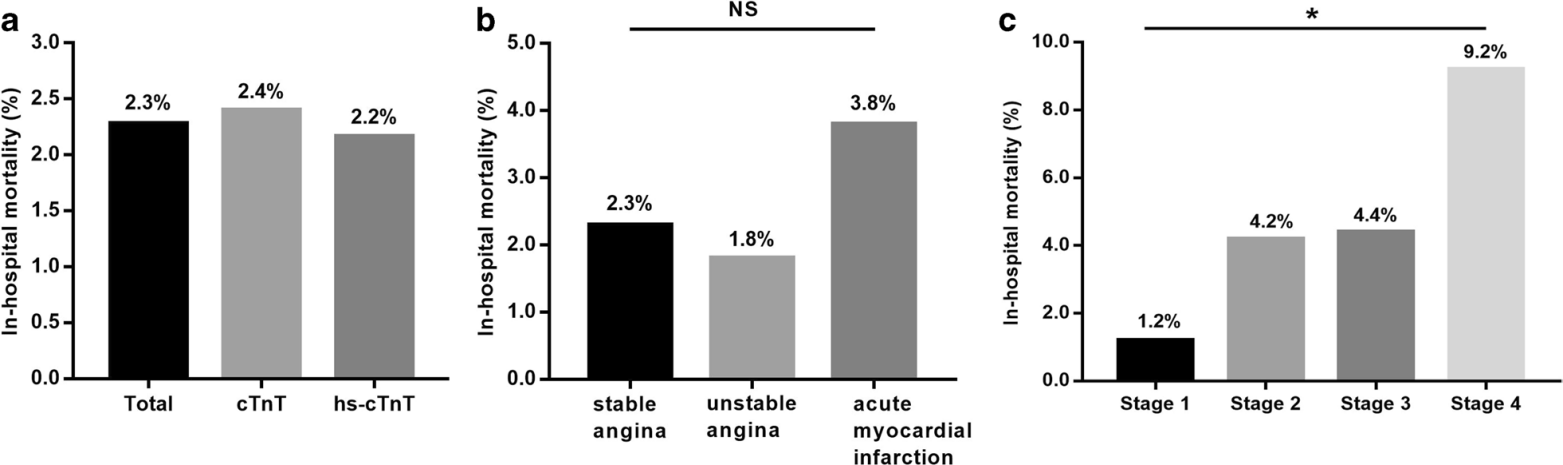
Overall and subsets in-hospital mortality. **a** In-hospital mortality in total cohort, cTnT group and hs-cTnT group. **b** In-hospital mortality in CAD subsets. **c** In-hospital mortality in myocardial injury subsets

Totally, 43 patients died in hospital, with a mortality of 2.30% (43/1887). In-hospital mortality rates in total cohort and different subsets were shown in Fig. 1. The in-hospital mortality in the AMI subset was 3.8% (Fig. 1b), and that at myocardial injury stage 4 was up to 9.2% (Fig. 1c).

### ROC curve

**Fig. 2:**
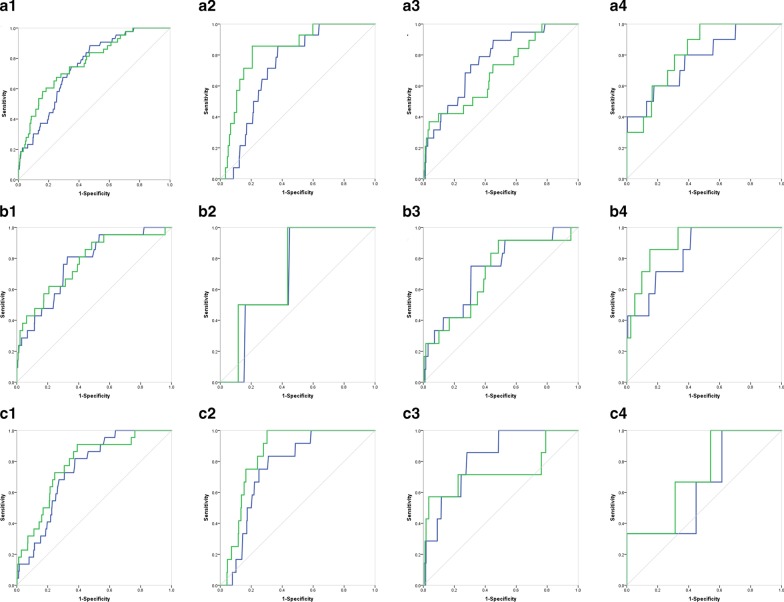
The ROC curves of the two risk evaluation systems in total cohort and subsets. **a** Total cohort and CAD subsets, 1. total cohort, 2. stable angina subset, 3. unstable angina subset, 4. AMI subset. **b** cTnT group and CAD subsets, 1. cTnT group, 2. stable angina subset, 3. unstable angina subset, 4. AMI subset. **c** hs-cTnT group and CAD subsets, 1. hs-cTnT group, 2. stable angina subset, 3. unstable angina subset, 4. AMI subset

The calibration and discrimination power of EuroSCORE II and the new risk evaluation system (EuroSCORE II integrating with cTnT) were shown in Table 3. In general, the new system outperformed in the total cohort and the subsets (Fig. 2). Especially in the AMI subset, the new system had obvious advantages in discrimination power (AUC 0.813 vs. 0.772, 0.906 vs. 0.841, and 0.715 vs. 0.646, respectively).

**Table 3:** The AUC of EuroSCORE II and new evaluation system in total cohort and subgroups

	H–L	AUC	95%CI (%)	*P*	H–L	AUC	95%CI (%)	*P*	H–L	AUC	95%CI (%)	*P*	H–L	AUC	95%CI (%)	*P*
	Total cohort (n = 1887)				SA group (n = 599)				UA group (n = 1028)				AMI group (n = 260)			
Euroscore II	0.121	0.746	0.684–0.808	0.000	0.223	0.727	0.641–0.813	0.004	0.987	0.765	0.670–0.860	0.000	0.193	0.772	0.619–0.925	0.004
New evaluation system	0.326	0.771	0.702–0.840	0.000	0.133	0.831	0.742–0.921	0.000	0.870	0.690	0.568–0.813	0.004	0.071	0.813	0.709–0.918	0.001

	H–L	AUC	95%CI (%)	*P*	H–L	AUC	95%CI (%)	*P*	H–L	AUC	95%CI (%)	*P*	H–L	AUC	95%CI (%)	*P*
	cTnT group (n = 971)				SA group (n = 243)				UA group (n = 567)				AMI group (n = 161)			
Euroscore II	0.407	0.769	0.676–0.862	0.000	0.384	0.699	0.494–0.904	0.332	0.959	0.725	0.587–0.863	0.070	0.437	0.841	0.717–0.966	0.002
New evaluation system	0.507	0.774	0.668–0.879	0.000	0.810	0.724	0.497–0.951	0.275	0.369	0.699	0.550–0.848	0.018	0.485	0.906	0.820–0.992	0.000

	H–L	AUC	95%CI (%)	*P*	H–L	AUC	95%CI (%)	*P*	H–L	AUC	95%CI (%)	*P*	H–L	AUC	95%CI (%)	*P*
	hs-cTnT group (n = 916)				SA group (n = 356)				UA group (n = 461)				AMI group (n = 99)			
Euroscore II	0.272	0.747	0.671–0.822	0.000	0.054	0.762	0.672–0.852	0.002	0.893	0.822	0.703–0.941	0.003	0.060	0.646	0.344–0.947	0.391
New evaluation system	0.405	0.782	0.694–0.869	0.000	0.132	0.849	0.794–0.905	0.000	0.664	0.736	0.490–0.982	0.032	0.699	0.715	0.456–0.975	0.206

AUC, area under receiver operating curve; H–L, Hosmer- Lemeshow statistic; CI, Confidence interval; cTnT, cardiac troponin T; hs-cTnT, high sensitivity cardiac troponin T; SA, stable angina; UA, unstable angina; AMI, acute myocardial infarction

### Net reclassification index

No significant differences in NRI were found in total cohort and the cTnT group (Table 4). It tended to be better NRI for the new system compared to EuroSCORE II. The new system outperformed EuroSCORE II in the hs-cTnT group (*P* = 0.04). Based on the types of CAD, there was a better NRI for the new system compared to EuroSCORE II in the unstable angina subset. No significant NRI was found in the other two subsets.

**Table 4:** Comparison of NRI for EuroSCORE II and new risk evaluation system

	NRI (%)	95% CI (%)	*P*
Total EuroSCORE II versus new evaluation system	− 11.24	− 25.10–2.62	0.06
Grouped by types of cTnT			
cTnT EuroSCORE II versus new evaluation system	− 9.85	− 32.39–12.71	0.20
hs-cTnT EuroSCORE II versus new evaluation system	− 19.44	− 38.17–-0.72	0.04
Grouped by types of CAD			
Stable angina EuroSCORE II versus new evaluation system	− 11.90	− 30.31–6.53	0.12
Unstable angina EuroSCORE II versus new evaluation system	− 24.54	− 49.19–0.12	0.04
AMI EuroSCORE II versus new evaluation system	17.20	− 1.99–36.39	0.95

NRI, net reclassification improvement; hs-cTnT, high sitivity cardiac troponin T; cTnT, cardiac troponin T; TC, TnT classification; CAD: coronary artery disease; AMI, acute myocardial infarction; CI, Confidence interval

### Bland–Altman analysis

**Fig. 3:**
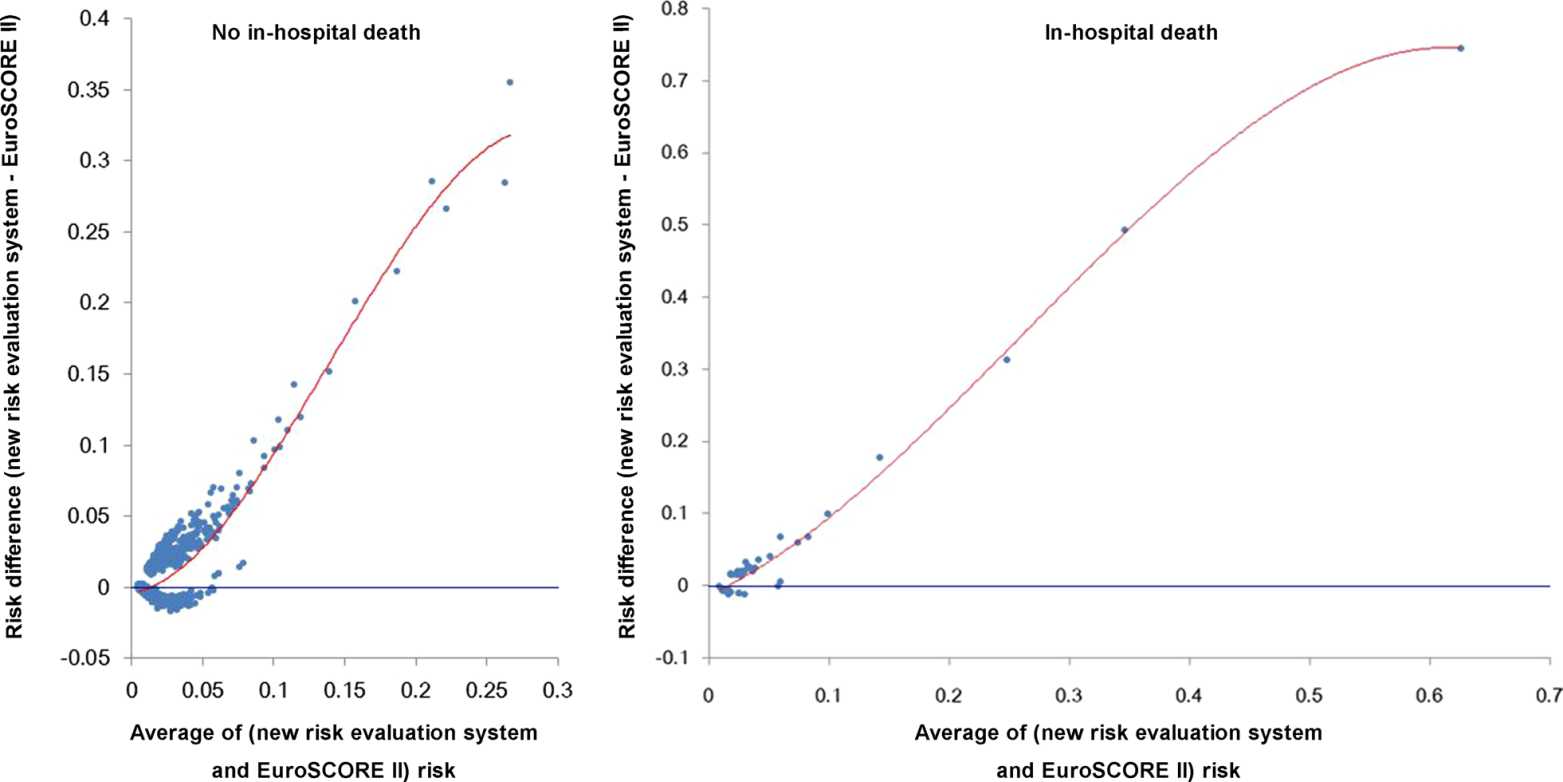
Bland–Altman plot of the new risk evaluation system and EuroSCORE II. The blue horizontal line means no difference between the two systems. **a** The plot compared the agreement in survivors. **b** The plot compared the agreement in dead patients in hospital

The agreement between the new risk evaluation system and EuroSCORE II was tested by Bland–Altman analysis (Fig. 3). Results showed the two systems were evaluated as a very good agreement. In the plot of survivors, most of the points were found in the agreement interval (97.9%). Similar result was found in the plot of death group (97.8%). Moreover, the new system reduced 65.7% of predictive mortality in the survivors and increased 65.1% of predictive mortality in the dead patients in hospital.

### Logistic regression analysis

The univariate logistic regression analysis showed that preoperative myocardial injury classification was a significant risk factor for in-hospital mortality in the total cohort (OR 2.049, 95%CI 1.542–2.722, *P < *0.001). It remained a significant risk factor for in-hospital mortality in the cTnT group and the hs-cTnT group (OR 3.564, 95%CI 2.105–6.035, *P < *0.001 and OR 1.963, 95%CI 1.322–2.915, *P* = 0.001). EuroSCORE II was also an independent risk factor for the postoperative mortality of OPCABG in both the total cohort and the cTnT group (Table 5). In the multivariate logistic regression models, myocardial injury classification and EuroSCORE II were also risk factors for in-hospital mortality (Table 6).

**Table 5:** Risk factors of in-hospital mortality by univariate logistic regression analysis

Variables	Total cohort (n = 1887)			cTnT group (n = 971)			hs-cTnT group (n = 916)		
	OR	95%CI	*P*	OR	95%CI	*P*	OR	95%CI	*P*
Age (y)	1.052	1.012–1.094	0.011	1.055	0.997–1.116	0.064	1.049	0.994–1.108	0.080
Female (n, %)	1.513	0.793–2.889	0.209	1.252	0.480–3.265	0.646	1.794	0.742–4.333	0.194
Weight (kg)	0.985	0.957–1.014	0.310	0.982	0.942–1.024	0.395	0.988	0.949–1.029	0.563
Height (cm)	0.984	0.945–1.024	0.422	0.965	0.911–1.022	0.223	1.001	0.946–1.060	0.961
NYHA classification (n, %)	2.425	1.562–3.766	0.000	2.552	1.329–4.901	0.005	2.321	1.286–4.191	0.005
Types of CAD (n, %)	1.231	0.776–1.951	0.377	2.256	1.129–4.509	0.021	0.720	0.363–1.427	0.347
Hypertention (n, %)	0.859	0.455–1.620	0.638	2.097	0.612–7.179	0.238	0.499	0.213–1.169	0.109
Diabetes (n, %)	1.450	0.781–2.694	0.239	1.512	0.630–3.626	0.354	1.409	0.584–3.398	0.446
Cerebrovascular disease (n, %)	0.210	0.050–0.871	0.032	0.292	0.067–1.261	0.099	0.000	0.000–0.000	0.997
Cr (μmol/l)	1.004	1.001–1.007	0.013	1.005	1.000–1.010	0.069	1.003	1.000–1.007	0.076
Ccr (ml/min/1.73 m^2^)	0.975	0.962–0.988	0.000	0.971	0.951–0.991	0.005	0.978	0.961–0.995	0.010
LVEF (%)	0.934	0.908–0.961	0.000	0.926	0.083–0.970	0.001	0.938	0.904–0.974	0.001
Number of diseased vessels (n)	1.071	0.539–2.126	0.845	2.542	0.426–15.184	0.306	0.757	0.362–1.585	0.461
Peripheral vascular disease (n, %)	4.296	1.470–12.556	0.008	3.466	0.770–15.608	0.105	5.86	1.256–27.349	0.024
Emergency operation (n, %)	6.096	2.602–14.284	0.000	11.143	2.943–42.187	0.000	4.398	1.427–13.559	0.010
Combined valvulardisease (n, %)	0.669	0.237–1.890	0.448	0.629	0.083–4.753	0.653	0.649	0.190–2.217	0.490
COPD (n, %)	0.805	0.109–5.956	0.831	0.000	0.000–0.000	0.998	1.283	0.167–9.834	0.811
Atrial flutter and fibrillation (n, %)	0.652	0.088–4.808	0.674	1.389	0.181–10.666	0.752	0.000	0.000–0.000	0.998
Pulmonary hypertension (n, %)	0.978	0.345–2.769	0.967	0.000	0.000–0.000	0.998	1.068	0.356–3.199	0.907
Previous PCI (n, %)	1.601	0.562–4.561	0.378	1.533	0.199–11.804	0.681	1.585	0.459–5.470	0.466
BMI (kg/m^2^)	0.960	0.870–1.060	0.424	0.988	0.860–1.135	0.864	0.934	0.810–1.077	0.348
Renal failure (n, %)	9.978	3.614–27.551	0.000	11.006	2.226–54.407	0.003	9.253	2.471–34.646	0.001
Euroscore II (%)	1.574	1.342–1.846	0.000	1.815	1.414–2.330	0.000	1.437	1.166–1.770	0.001
Myocardial injury classification (n, %)	2.049	1.542–2.722	0.000	3.564	2.105–6.035	0.000	1.963	1.322–2.915	0.001

hs-cTnT, high sitivity cardiac troponin T; cTnT, cardiac troponin T; NYHA, New York Heart Association; BSA, body surface area; Cr, creatinine; Ccr, creatinine clearance rate; LVEF, left ventricular ejection fraction; COPD, chronic obstructive pulmonary disease; PCI, percutaneous coronary intervention; BMI, body mass index; EuroSCORE, European system for cardiac operative risk evaluation

**Table 6:** Risk factors of in-hospital mortality by multivariate logistic regression analysis

Variables	Total cohort (n = 1887)			cTnT group (n = 971)			hs-cTnT group (n = 916)		
	OR	95%CI	*P*	OR	95%CI	*P*	OR	95%CI	*P*
Age (y)	1.021	0.973–1.071	0.403	1.001	0.924–1.805	0.977	1.032	0.967–1.101	0.344
Female (n, %)	1.123	0.536–2.353	0.758	0.883	0.296–2.640	0.824	1.595	0.543–4.682	0.396
NYHA classification (n, %)	1.540	0.936–2.535	0.089	1.198	0.530–2.659	0.677	1.870	0.933–3.749	0.078
Cerebrovascular disease (n, %)	0.179	0.042–0.774	0.021	0.202	0.044–0.920	0.039	0.000	0.000–0.000	0.996
Ccr (mL/min/1.73 m^2^)	0.995	0.977–1.012	0.551	0.992	0.965–1.020	0.567	1.002	0.979–1.026	0.851
LVEF (%)	0.959	0.977–1.012	0.551	0.953	0.893–1.016	0.140	0.954	0.910–1.000	0.049
Peripheral vascular disease (n, %)	3.099	0.954–10.066	0.060	2.176	0.398–11.903	0.370	3.579	0.607–21.117	0.159
Emergency operation (n, %)	3.256	1.115–9.506	0.031	0.549	0.029–10.402	0.689	5.232	1.449–18.885	0.012
Renal failure (n, %)	2.016	0.443–9.170	0.364	0.627	0.040–9.701	0.738	5.681	0.793–40.713	0.084
Myocardial injury classification (n, %)	1.491	1.049–2.119	0.026	2.421	1.030–5.689	0.042	1.761	1.102–2.814	0.018
EuroSCORE II (%)	1.310	1.045–1.643	0.019	1.544	1.015–2.349	0.043	1.273	0.879–1.844	0.201
Hypertension (n, %)	0.823	0.406–1.669	0.589	1.889	0.502–7.108	0.347	0.482	0.185–1.256	0.135
Diabetes (n, %)	1.259	0.634–2.498	0.511	1.709	0.653–4.475	0.275	0.871	0.310–2.444	0.793
Combined valvulardisease (n, %)	0.388	0.125–1.199	0.100	0.273	0.031–2.392	0.241	0.326	0.079–1.345	0.121
BMI (kg/m^2^)	0.996	0.884–1.123	0.949	0.992	0.830–1.184	0.925	0.984	0.823–1.176	0.859

hs-cTnT, high sitivity cardiac troponin T; cTnT, cardiac troponin T; NYHA, New York Heart Association; BSA, body surface area; Ccr, creatinine clearance rate; LVEF, left ventricular ejection fraction; EuroSCORE, European system for cardiac operative risk evaluation; BMI, body mass index

### Decision curves analysis

**Fig. 4:**
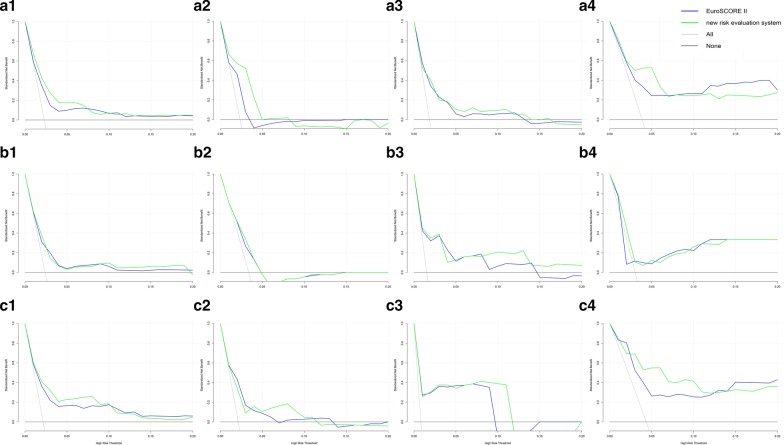
DCA for assessing the clinical benefits of the two risk evaluation systems in total cohort and subsets. The gray line represents the net benefits of providing surgery for all patients, assuming that all patients would survive. The black line represents the net benefits of surgery to no patients, assuming that none would survive after operation. The blue and green lines stand for the net benefits of applying surgery to patients according to EuroSCORE II and the new system respectively. **a** total cohort and CAD subsets. 1. total cohort, 2. stable angina subset, 3. unstable angina subset, 4. AMI subset. **b** cTnT group and CAD subsets, 1. cTnT group, 2. stable angina subset, 3. unstable angina subset, 4. AMI subset. **c** hs-cTnT group and CAD subsets, 1. hs-cTnT group, 2. stable angina subset, 3. unstable angina subset, 4. AMI subset

The clinical benefits of EuroSCORE II and the new system in predicting in-hospital mortality were calculated by DCA. The net benefits of the two systems for predicting in-hospital mortality in the total cohort and subsets were shown in Fig. 4. In general, the new system was no worse than EuroSCORE II. Especially in the total cohort, the stable angina subset, the AMI subset, the cTnT group and the cTnT AMI subset, the new risk evaluation system outperformed EuroSCORE II with better net benefits.

## Discussion

### Principal findings

Consistent with our hypothesis, the increase of preoperative cTnT or hs-cTnT was an independent risk factor for postoperative mortality in patients undergoing OPCABG. When EuroSCORE II was integrated with the myocardial injury classification, the ability to predict the outcomes after OPCABG can be improved. Especially for patients with preoperative AMI, integration with myocardial injury classification can significantly improve the discrimination power of EuroSCORE II, which is of great clinical significance for high-risk patients. According to our literature review, it is the first time to report the combination of cTnT in improving the prediction ability of EuroSCORE II for OPCABG patients in China.

### Cause analysis

EuroSCORE was based on the surgical data of more than 10,000 patients who underwent cardiac surgery in 8 European countries in 1995 [12], and has been used worldwide for decades. With the prolonging of time, EuroSCORE was also advancing and constantly updated. In 2012, the EuroSCORE research team proposed a new system—EuroSCORE II [13]. However, with the continuous advancement of surgical techniques and the increasing amount of cardiac surgery worldwide, EuroSCORE II will overestimate or underestimate the risk of death in patients undergoing cardiac surgery [14–19]. The performance of the EuroSCORE II was poor among patients with the lower predicted mortality [20]. Moreover, our previous research considered that EuroSCORE II underestimated the risk of cardiac surgery for the Chinese population, especially for CABG patients [21, 22]. Continuing to use the model may mislead the judgment of clinicians and even will harm the interest of patients. In addition, EuroSCORE II was designed to predict the perioperative mortality [13] and was inappropriate to predict postoperative mid- and long-term risks. Because many factors that can affect the long-term mortality [23, 24], for in-hospital mortality, the predictive ability of EuroSCORE II may be improved theoretically when it integrates with some new risk factors.

cTnT is one of the cardiac calmodulin subunits and a specific marker of myocardial injury [25, 26]. When myocardial cells damaged, with the rupture of cell membrane structure, cTnT quickly enters circulation [27, 28]. The continuous progress of the production technology has continuously improved the detection level of cTnT [29]. Nowadays, the fourth generation of hs-cTnT has been widely used in clinic. Novel highly sensitive assays for hs-cTnT can detect troponin concentration 10 times lower than the standard assays [30].

Literature has confirmed that the increase of cTnT is a strong risk factor of recent adverse cardiovascular events and is also applicable to patients undergoing cardiac intervention and surgery [31–34]. As reported, cTnT before percutaneous coronary intervention can provide better predictive value for postoperative outcomes [35]. In this study, both univariate and multivariate analyses found that cTnT was an independent risk factor for in-hospital mortality.

EuroSCORE II contains 18 important perioperative risk factors, but does not involve any factor of myocardial injury [13]. Nowadays, cTnT test, a routine in the daily work of CABG [5], is indispensable for patients with myocardial ischemia. Therefore, we tried to integrate preoperative cTnT information into EuroSCORE II and found that its predictive power was improved after integrating with myocardial injury classification.

One of the important functions of risk evaluation systems is to distinguish high-risk patients before operation, which is of great significance and importance to the allocation of medical resources and the communication between doctors and patients’ families. Bland–Altman analysis showed good agreement between the two risk evaluation systems for patients who died or survived after operation. The new risk evaluation system in the surviving patients was scored lower than EuroSCORE II, while was scored higher in the dead patients. These results illustrate the new system can replace EuroSCORE II, and has a higher ability to distinguish high-risk patients. Generally, the risk of operation in AMI patients is higher than of other patients [36]. Subset analysis found the new system significantly improved the predictive power in the AMI subset. The discriminative power of the new system was 0.813, 0.906 and 0.715 respectively. No matter in total cohort or in cTnT group or hs-cTnT group, they all showed the same trends in the AMI patients. In general, after integrating myocardial injury variable, EuroSCORE II could improve the ability to identify high-risk patients before cardiac surgery, which was beneficial to personalized treatment and optimization of medical resources.

Interestingly, the discriminative power of the new risk evaluation system was not as good as EuroSCORE II in the unstable angina subset. The reason may be that the degree of myocardial injury in patients with unstable angina was very different, which affected the contribution of cTnT to the prediction.

### Limitations

There are some limitations in our study. Firstly, it was a retrospective observational study in a single medical center, and the sample size was small. Secondly, surgical practice, anesthesia and postoperative care changed over the study period, which can lead to selection bias. Thirdly, considering the influence of renal dysfunction on cTnT [37, 38], we excluded such patients, which may also cause selection bias. Fourthly, we converted cTnT from a continuous variable to a categorical variable, which will lose some information.

## Conclusions

Both myocardial injury classification and EuroSCORE II are independent risk factors of in-hospital mortality in OPCABG patients. The inclusion of cTnT can enhance the predictive ability of EuroSCORE II, especially in patients with a recent history of AMI.

## Data Availability

If readers need complete original data, they can contact the corresponding author to obtain it.

## Abbreviations

EuroSCORE II: European system for cardiac operative risk evaluation II
cTnT: Cardiac troponin T
OPCABG: Off-pump coronary artery bypass grafting
CABG: Coronary artery bypass grafting
hs-cTnT: High-sensitivity cardiac troponin T
UNL: Upper normal limit
NYHA: New York heart association
Ccr: Creatinine clearance rate
LVEF: Left ventricular ejection fraction
BMI: Body mass index
H–L: Hosmer–Lemeshow
ROC: Receiver’s operating characteristic
DCA: Decision curves analysis
NRI: Net reclassification index
CAD: Coronary heart disease
AMI: Acute myocardial infarction
